# Cancer bioimaging using dual mode luminescence of graphene/FA-ZnO nanocomposite based on novel green technique

**DOI:** 10.1038/s41598-022-27111-z

**Published:** 2023-01-02

**Authors:** Wessam Wanas, Samir Ali Abd El-Kaream, Shaker Ebrahim, Moataz Soliman, Marwa Karim

**Affiliations:** 1 Department of Materials Science, Institute of Graduate Studies and Research, Alexandria University, P.O. Box 832, Alexandria, Egypt; 2 Department of Applied Medical Chemistry, Medical Research Institute, Alexandria University, Alexandria, Egypt; 3 Physics Department, Faculty of Science, Alexandria University, Moharram Bek, P.O. Box 21511, Alexandria, Egypt

**Keywords:** Materials science, Nanoscience and technology

## Abstract

Graphene based nanomaterials are explored in the field of cancer bioimaging and biomedical science and engineering. The luminescent nanostructures with a low toxicity and high photostability can be used as probes in bioimaging applications. This work is aimed to prepare graphene/folic acid-zinc oxide (GN/FA-ZnO) nanocomposite with dual-mode emissions (down-conversion and up-conversion) to be used in cancer bioimaging. The dual mode emissions offer long luminescence lifetime, multicolor emissions detected by the naked eyes after excitation and narrow band absorption and emission spectra. ZnO nanospheres and nanorods structures were prepared using co-precipitation technique and were conjugated with FA to separate the bulk graphite layers electrostatically into GN. The optical, morphological, surface charge and structural properties of the prepared nanostructures were investigated and discussed using different characterization techniques such as UV–visible spectroscopy, photoluminescence (PL) spectroscopy, scanning electron microscope (SEM), high resolution transmission electron microscope (HRTEM), Zeta potential, Raman spectroscopy, X-ray photoelectron spectroscopy (XPS), X-ray powder diffraction (XRD), and Fourier transform infrared (FTIR). GN/FA-ZnO nanocomposites were injected into Swiss albino mice implanted with Ehrlich Tumor and the bioimaging was investigated using photon imager and digital camera. The results showed clear fluorescence and confirmed that the green design of GN/FA-ZnO nanocomposite with targeting behavior was capable of selective bioimaging of the tumor. This study presented a novel dual mode emission nanocomposite for tumor targeting and is a promising strategy for the fabrication of a new design of spectral encoding.

## Introduction

Nanotechnology is defined as a multidisciplinary approach at the nanoscale for fabricating and developing materials that have many applications including catalysis^1–3^, industrial applications^4,5^, tissue engineering^6^, biomedical investigations^7–9^ and sensing technology^10–12^. Nanomedicine can be specified as a science area that includes nanotechnology with diagnostic molecules or drugs to enhance the capability to target tissues or specific cells. The applications of nanotechnology in medicine include diagnosis, imaging and drugs delivery due to the small size of nanomaterials that enable to overpass different barriers of in vivo biological tissues including blood–brain barrier and ion channels that will help in the treatment of various diseases^13^.

Imaging contrast parameters-based nanotechnology are being updated every day and are specifically presented an ability for detection the tumors in vivo compared with conventional scanning devices^14–16^. Furthermore, the platforms of nanoscale imaging have novel patterns with a lower health risk and a higher penetration^17^. Up-conversion nanostructures are categorized as new class of luminescent nanomaterials, which have more advantages than traditional fluorophores, such as photostability and high signal-to-noise ratio. The excitation wavelengths of these structures in the range of near-infrared have low photodamage to biological samples and deep tissue penetration^18–20^.

Metal oxide nanoparticles (NPs) such as ZnO NPs have a remarkable achievement in the biomedical field due to their luminescence efficiency^21^ and anticancerous properties^22^. ZnO NPs doped with HO^3+^ and Yb^3+^ prepared using sol–gel method has two intense emission bands in the up-conversion emission spectra, 542 nm green and 660 nm red^23^. Functionalized ZnO nanostructures with FA achieved stable and dispersed aqueous ZnO NPs^24^. FA is one of the vitamin B family and is an important for the synthesis and repair of DNA and other genetic materials, and it is necessary for cells to divide. It is essential for cellular pathways biosynthesis and intracellular activity^25^. Furthermore, FA has an ability to control NPs sizes due to its surface density^26,27^. In recent years, to promote the photoluminescent properties of the nanostructures, the combination of FA with carbon-based nanomaterials is carried. The different carbon nanostructures of different allotropes as graphene, diamond, nanotubes and fullerenes have advantages in biological studies^28,29^. Graphene is an extremely light material, highly transparent to visible light and has the toughest and hardest crystal structure of the known materials. Functionalization of GN allows for the tuning of the ability of the monolayers to be processed in solution and prevent agglomeration^30^.

The interaction between ZnO NPs and GN improves the charge transfer of electrons, electrical conductivity and optoelectronic property that enhances ultrafast nonlinear optical transferring capability for bioimaging applications^31^. Hu et al. prepared GO-FA-ZnO via chemical precipitation method for targeting photodynamic therapy using visible light. The interaction between ZnO NPs and GO enhanced the photodynamic activity with a low cytotoxicity^32^. However, the results showed that GO was produced, they did not use concept of up-conversion technique. The aim of this work is to synthesis and characterize dual mode luminescence of GN/FA–ZnO nanocomposite by one step liquid phase exfoliation using sonication under different conditions to be used for cancer bioimaging. Effect of dual-mode emissions of GN/FA–ZnO nanocomposite using down-conversion and up-conversion concepts on PL properties is investigated. In addition, the cancer bioimaging is evaluated by injection of GN/FA-ZnO nanocomposite into Swiss albino mice implanted with Ehrlich Tumor.

## Materials and methods

### Chemicals and materials

Zinc sulphate and graphite powder were commercially purchased from Fisher Chemical with purity of 99.0%. Sodium hydroxide and ethanol were purchased from Across Company with purity of 99.8%. Folic acid was purchased from Molebase with purity of 99.98%.

### Synthesis of GN/FA-ZnO nanocomposite

Different ZnO nanostructures were produced by precipitation technique where the solution of sodium hydroxide (0.2 M) was added to zinc sulfate solution (0.1 M) with a strong stirring for 12 h. Ethanol and deionized water were used to wash the obtained precipitate and this precipitate was dried and ground to a fine powder. The final product was annealed for 2 h at different temperatures of 100, 300, 500, and 700 °C^33^. FA-ZnO NPs were prepared using different concentrations of FA (5 × 10^–4^, 1 × 10^–3^, 2 × 10^–3^ and 3 × 10^–3^ M) and spherical ZnO (0.01 M) with a continuous stirring for 2 h. FA-ZnO NPs with a concentration of 0.001 M was used directly to prepare GN/FA-ZnO nanocomposite from bulk graphite through one step method. Graphite with different molar ratios of 5, 10, 20 and 100% was added into FA-ZnO solution and sonicated for 3 h to produce GN/FA-ZnO nanocomposites. After sonication, the obtained dark dispersion was stored and stand overnight for allow the large particles to sediment. To ensure removing any large flakes, the solution was centrifuged at 4000 rpm for 15 min to produce a homogeneous nanocomposite of GN/FA-ZnO suspension. The use of FA is not only for electrostatically separation of bulk graphite but also it can be used as a ligand for cancer cell receptors and used to detect tumor cells due to the presence of folate receptors in tumor cells. The novelty of this work was arisen from the preparation of the GN/FA-ZnO nanocomposite with high content of graphene sheets and its ability to achieve dual mode luminescence, sensitivity and selectivity properties for cancer bioimaging.

### Animals and Ehrlich tumor cells

Ehrlich ascites carcinoma (EAC) is referred to as an undifferentiated carcinoma, rapid proliferation, also does not have tumor-specific transplantation antigen. Ehrlich Tumor is mammary gland in origin cancer representative for breast cancer model easily implanted tumor and express folic acid receptor which is target point for the bioimaging^34^. Swiss albino mice were obtained with 20 ± 5 g weight and age of 8 weeks from National Cancer Institute, Cairo University, Egypt. EAC tumor cells were diluted in 0.9% saline and subcutaneously inoculated on the abdominal region of the mice. The animals were preserved under the light with water and diet in plastic cages. When the tumor diameter was grown to about 1 mm^3^ after 10 days of inoculation, the mice were applied to bioimaging. There were three groups of mice having tumor:

**Control group (I): (a)** Mice without injection (n = 6) for bioimaging at 400 nm excitation wavelength.

**(b)** Mice without injection (n = 6) for bioimaging at 630 nm excitation wavelength.

**Group (II):** Mice injected with GN/FA-ZnO nanocomposite and excited with 400 nm (n = 6).

**Group (III):** Mice injected with GN/FA-ZnO nanocomposite and excited with 630 nm (n = 6).

The main objective of studying biological imaging was to determine the performance of dual mode luminescence imaging for GN/FA-ZnO nanocomposite. Before the process of bioimaging, mice were anaesthetized using isoflurane. After 30 min of intraperitoneal injection of 25 ul per mouse of concentration 10% GN/FA-ZnO nanocomposite that was dissolved in phosphate buffer saline, the dual mode bioimaging of mice were carried out.

#### Ethics statement

Experimental procedures, animal handing and sampling followed the Guide for the Care and Use of Laboratory Animals, 8th edition (National Research Council, 2011) were approved by Research ethical Committee (Appendix 2; Guide Principles for Biomedical Research Involving Animals, 2011, No: AU0122018131) of the Medical Research Institute, Alexandria University (Alexandria, Egypt). This study was reported in accordance with ARRIVE guidelines (https://arriveguidelines.org).

### Characterization techniques

The spectra of absorption and photoluminescence (PL) were scanned with UV–vis spectrophotometer (Thermo-Evolution 600) and PL spectrophotometer (Perkin Elmer LS-55), respectively. A Fourier transform infrared (FTIR) (Perkin -Elmer BXII) spectrophotometer was used to show the effect of annealing on different ZnO nanostructures and determine various vibrational modes presented in ZnO nanostructure. Scanning electron microscope (SEM) (JOEL (JSM 6360LA)) and high-resolution transmission electron microscopy (HRTEM) (JEOL (JEM-2100 LaB6)) were applied to determine the size and homogeneity of the nanostructures. Confocal Raman spectrophotometer (Wltec, 300R alpha) was used to determine structure and type of the graphitic structure obtained. X-ray photoelectron spectroscopy (XPS) (Thermo scientific K-Alha) with Al-Kα monochromator with an energy range up to 4 keV was used to analyze chemical states and binding energies of chemical bonds in the nanocomposite. X-ray powder diffraction (XRD) (Brucker-AXS D8 Discover) was used to determine the crystal structure and crystallite size. Zeta potential (ZP) (NanoZS/ZEN3600 Zetasizer, Malvern) was used to measure surface charges onto the particles. Investigating the mice bioimaging was performed by photon imager with 400 nm excitation wavelength (down-conversion effect). Laser with excitation wavelength 630 nm (model LAS 50- Hi-Tech fysiomed) was used in a dark room to observe luminescence and a digital camera was used to record the results (up-conversion effect).

## Results and discussion

### Absorption properties of ZnO NPs

**Figure 1:**
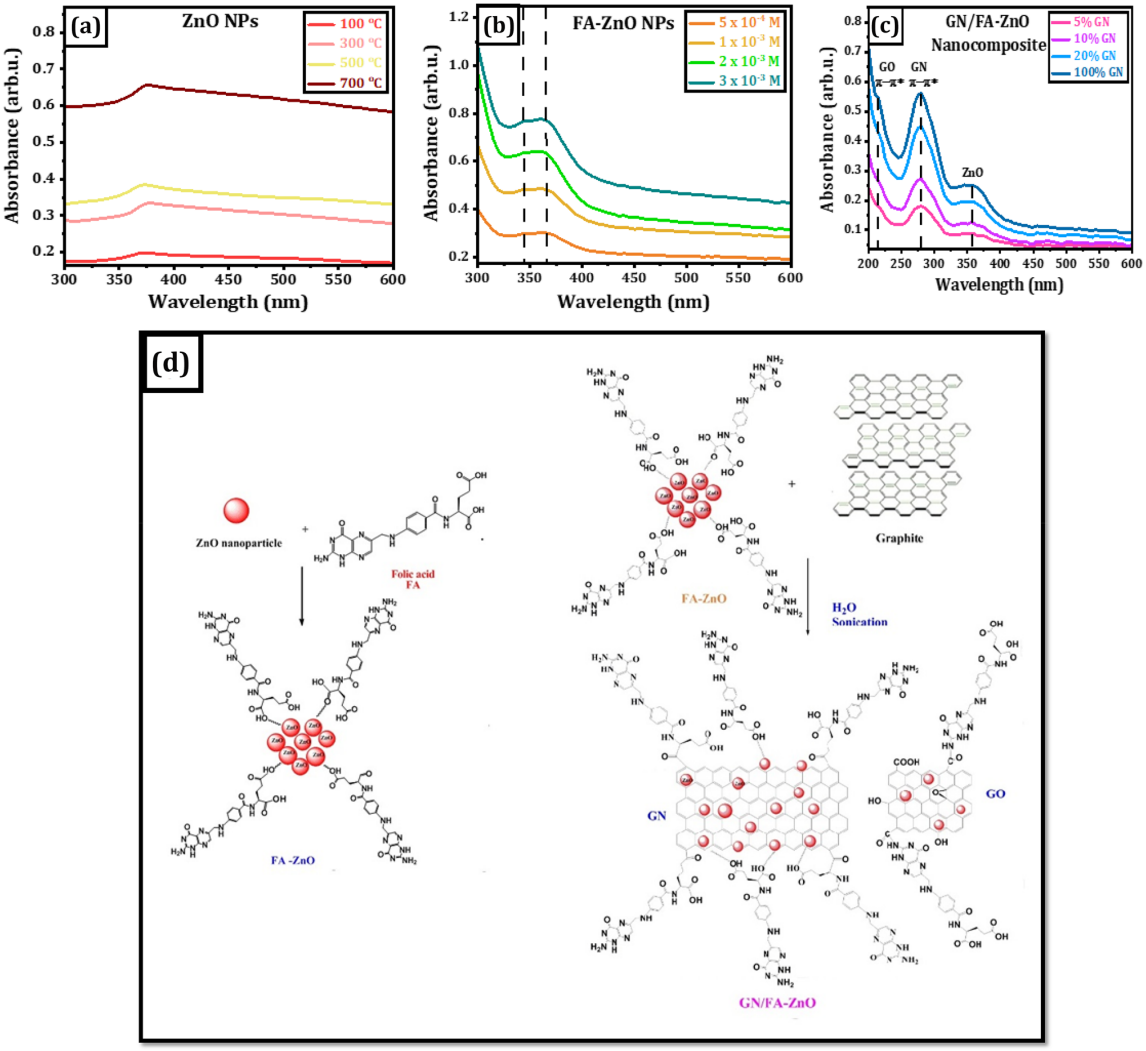
UV–vis spectra of different nanostructures: (**a**) ZnO NPs at different annealing temperatures, (**b**) FA-ZnO NPs prepared with different concentrations of FA, (**c**) GN/FA-ZnO nanocomposite at different ratios of GN and (**d**) Schematic diagram GN/FA-ZnO nanocomposite.

The absorption spectra are carried out to study optical properties of different ZnO nanostructures. The absorption spectra of ZnO nanostructures with different annealing temperatures have different peaks at 373, 378, 375 and 377 nm corresponding to annealing temperatures of 100, 300, 500 and 700 °C, respectively as shown in Fig. 1a. The spectra reveal characteristic absorption peaks due to the transition of electrons from the valence band to the conduction band (O_2p_ → Zn_3d_)^35^. It is observed that there are slightly redshifts of these peaks, and the absorption intensities are enhanced due to the variation of particles sizes. In addition, raise the annealing temperature from 100 to 700 °C results in the conversion of nanorods ZnO to nanospheres^35^.

The effect of FA concentrations (5 × 10^–4^, 1 × 10^–3^, 2 × 10^–3^ and 3 × 10^–3^ M) on the absorption spectra of FA-ZnO NPs is displayed in Fig. 1b. It is noted that there are two small absorption peaks at 344 and 360 nm. The blue shift of these peaks can be explained based on the reduction in the particle size of ZnO NPs^31^. These two peaks originate from the presence of FA-ZnO NPs with different sizes. The absorption spectra of GN/FA-ZnO nanocomposite prepared with 1 × 10^–3^ FA shown in Fig. 1c have new high peaks at 279 nm corresponding to the different GN ratios. This high absorption peak is assigned π–π* transitions of C–C aromatic bonds of GN^36^. There is another small absorption peak at 357 nm of ZnO NPs and a small shoulder at 218 nm due to π–π* GO. The synthesis process of GN/FA-ZnO nanocomposite is schematically shown in Fig. 1d. Firstly, FA is conjugated to ZnO via the carboxylate groups electrostatically. Secondary, FA-ZnO NPs with graphite under sonication exfoliates the graphite layers to form GN with a low amount of GO via imide linkage. It is proposed that FA-ZnO NPs are inserted between layers of GN. It is concluded that GN/FA-ZnO nanocomposite is prepared with high concentration of GN with low content of GO.

### Photoluminescence performance

#### Down-conversion properties

**Figure 2:**
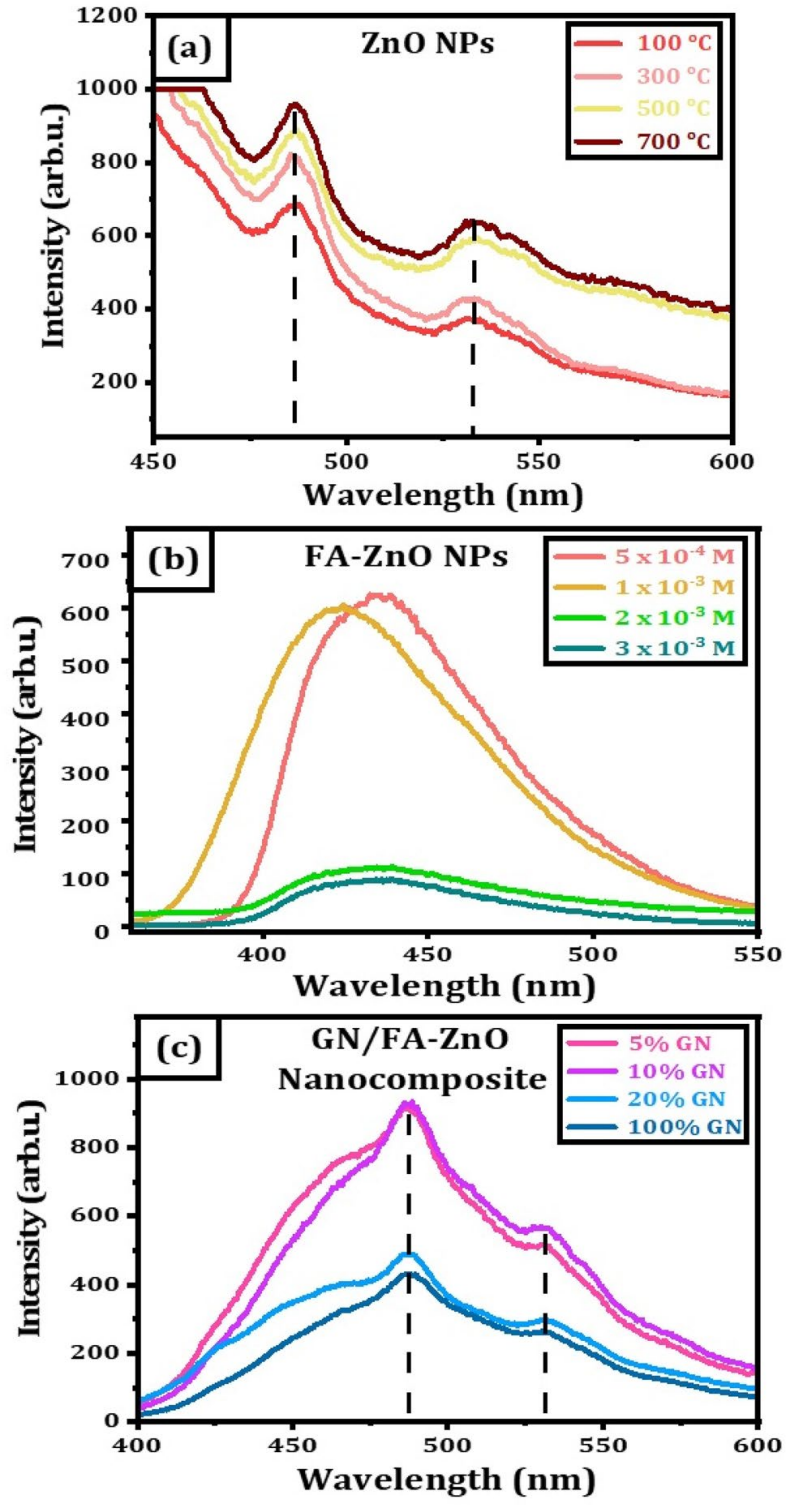
PL spectra (**a**) ZnO nanostructures at different annealed temperature, (**b**) FA-ZnO nanoparticles at different concentrations of folic acid; (**c**) GN/FA-ZnO nanocomposite prepared at different contents of GN.

Photoluminescence is a process in which a molecule absorbs a photon in the visible region, exciting one of its electrons to a higher electronic excited state, and then radiates a photon as the electron returns to a lower energy state. Using wavelength of 365 nm as the excitation wavelength, luminescence spectra of ZnO NPs prepared at annealing temperatures of 100, 300, 500 and 700 °C are presented in Fig. 2a. The spectra have two characteristic emission peaks. The first high emission peak at 486 nm is a blue defect luminescence due to the oxygen vacancies in ZnO lattice or originating from the recombination of free excitons through an exciton-exciton collision process^37–39^. The second small emission peak at 533 nm is a green emission ascribed to electrons transitions from the conduction band to valence band^38^. PL spectra of FA-ZnO NPs annealed at 700 °C and prepared with different FA concentrations are shown in Fig. 2b. The dispersed solution of FA-ZnO NPs displays broad emission peaks in the region of 380–580 nm. The spectra show a vital change of single blue emission peaks compared to pristine ZnO NPs due to charge transfer reaction^27^. For FA-ZnO NPs prepared with 5.0 × 10^–4^ and 1.0 × 10^–3^ M FA, the emission peaks appear at ~ 432 and 447 nm, respectively. The spectrum shows a blue shift and a lower intensity with increasing FA concentration to 2.0 × 10^–3^ M and 3.0 × 10^–3^ M. This is attributed to the surface states passivation of ZnO NPs by the high concentration of FA.

Effect of GN ratios on PL spectra of GN/FA-ZnO nanocomposite with different contents of GN is presented in Fig. 2c. The spectra have two high and small emission peaks at 485 and 538 nm, respectively. The high emission peak at 485 nm is independent of the wavelength at different contents of GN due to the electron transition from superficial donor level produced by interstitial Zn to superficial acceptor level produced by Zn vacancies. On the other hand, small PL peaks at 538 nm result in the transition from deep donor levels by the oxygen vacancies to valence band^40^. Increasing the concentration of GN, the emission intensity is obviously decreased and quenched due to the electron transfer from ZnO conduction band to GN^41^. Nanocomposite of 10% GN has the maximum activity and enhances the photodynamic activity for sensing property.

#### Up-conversion properties

**Figure 3:**
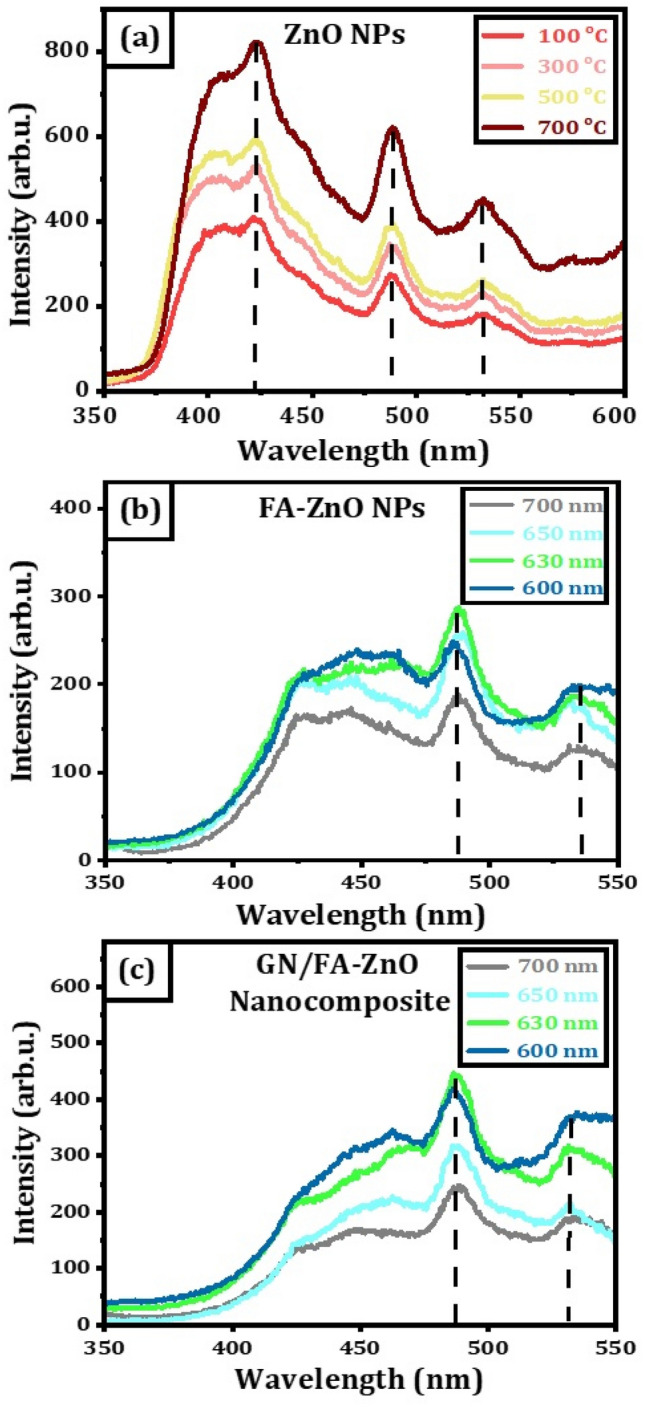
Up-conversion spectra (**a**) ZnO nanostructures at different annealed temperatures, (**b**) FA-ZnO NPs at different excitation wavelengths; (**c**) 10% GN/FA-ZnO nanocomposite prepared at different excitation wavelengths.

Up-conversion luminescence of the ZnO nanostructures is obtained using 630 nm as excitation wavelength. The spectra reveal four characteristic emission peaks with wavelengths of 395–418–482–527, 399–420–484–529, 400–422–485–530 and 402–425–488–532 nm corresponding to annealing temperatures of 100, 300, 500 and 700 °C, respectively as shown in Fig. 3a. The emission peaks are existed from 395 to 402 nm. The emission peaks appear in the blue zone from 418 to 425 nm and the other blue emission peaks from 482 to 488 nm. The green emission peaks are located from 527 to 532 nm. It is noted that the intensity of up-conversion luminescence of ZnO nanostructures increases with increasing the annealing temperature due to improving the crystallinity, leading to reduce both the internal lattice defects and non-radioactive relaxation^23^.

Up-conversion luminescence of FA-ZnO NPs is measured using different excitation wavelengths as shown in Fig. 3b. FA-ZnO NPs have up-conversion spectra in the region of 350–650 nm. The spectra reveal five emission peaks at different excitation wavelengths. The emission peaks from 425 to 487 nm are existed in the blue zone and emission peaks from 530 to 533 nm are located in the green region. Up-conversion luminescence spectra of GN/FA-ZnO nanocomposite with 10% GN are studied at different excitation wavelengths. The spectra have different characteristic emission peaks of GN/FA-ZnO nanocomposite at wavelengths of 423–460–484–531; 425–462–486–529, 423–456–485–528, and 421–444–485–528 corresponding to 700, 650, 630 and 600 nm, respectively as displayed in Fig. 3c. As a result of using different near infrared excitation wavelengths, blue and green emissions are produced. The blue emission is located from 421 to 486 nm and green emission is located from 528 to 531 nm. The up-conversion emission peaks in ZnO nanostructures can be originated via different mechanisms including the process of two-photon absorption (TPA) and/or two-step two-photon absorption (TS-TPA)^42–44^. Excitation in both processes is achieved by absorption of two photons with an intermediate state. TPA can be produced by a virtual state which needs high excitation power. The source of the impurities or defects in ZnO nanostructures is responsible for the process of TS-TPA and the true position of energy corresponding to intermediate stats.

It is concluded that GN/FA-ZnO nanocomposite shows both down and up-conversion properties. In down-conversion emission, the nanocomposite is excited by photons with short wavelength, and emits photons with longer wavelength. On the other hand, in up-conversion emission, the nanocomposite is excited by photons with long wavelength and emits photons with shorter wavelength. Up-conversion fluorescence bioimaging with an excitation in the near-infrared has been used for imaging of biological cells due to the absence of photo-damage to living organisms and high light penetration depth in biological tissues.

### FTIR of ZnO nanostructures

**Figure 4:**
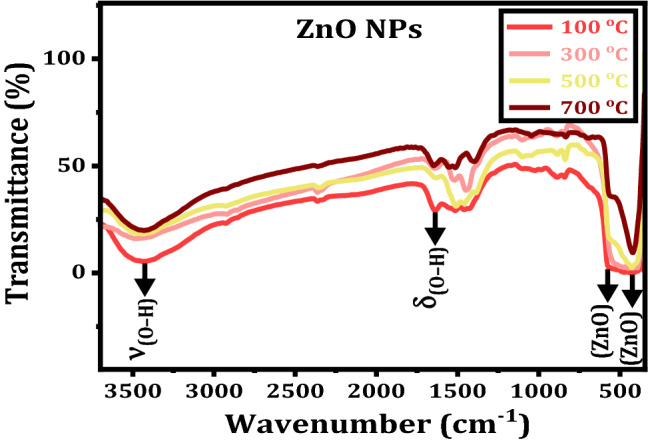
FTIR spectra of the ZnO NPs annealed at different temperatures.

FTIR spectroscopic technique is used to detect the functional groups and provides more information about the chemical composition of materials. To study the effect of annealing temperature on vibrational modes of ZnO NPs, FTIR spectra are displayed in Fig. 4. There are various characteristic absorption bands in the region of 350–4000 cm^−1^. The wide bands around 3550 and 1660 cm^−1^ are assigned to stretching and bending vibrational modes of hydroxyl groups (O–H), respectively. The peaks at 350 and 500 cm^−1^ are corresponded to vibrational modes of Zn–O^39,45^. It is noted that the intensity of bands is enhanced with increasing annealing temperature and is directly related to Zn–O bonds number^46^. The sharpness of ZnO peak with the increase of annealing temperature is due to reduction of O–H mode^47^.

### Structural analysis of GN/FA-ZnO nanocomposite

**Figure 5:**
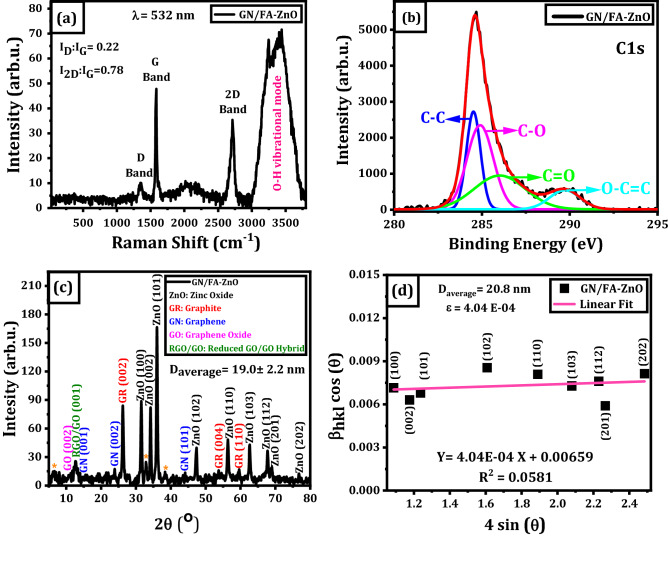
(**a**) Raman spectra of GN/FA-ZnO nanocomposite, (**b**) XPS analysis of GN/FA-ZnO nanocomposite, (**c**) XRD pattern with the presented phases in the nanocomposite and (**d**) W–H plot from the XRD pattern.

Raman spectroscopy is a simple and powerful characterization tool to determine structure, type of graphitic structures and number of graphene layers. Here, Raman spectra are detected under excitation laser (λ = 532 nm) for GN/FA-ZnO nanocomposite as displayed in Fig. 5a. Raman spectra exhibit three characteristic peaks at 1349.8, 1580.9 and 2715.2 cm^−1^. The sharp order G-band at 1580.9 cm^−1^ results from the first order scattering of E_2g_ phonon characteristic of sp^2^-hybridization of C–C bonds (i.e., in-plane vibrational mode of C–C bonds)^48^. On the other hand, the weak D-band at 1580.9 cm^−1^ originates from the breathing oscillation of k-point phonons with A_1g_ symmetry^49^. The presence of D-band indicates defects and disorders in GN structure due to the oxidation of the carbon bonds^49,50^. In addition, 2D-band at 2715.2 cm^−1^ resembles a second order overtone of the in-plane vibration mode of the D-band. Thus, the presence G, D, and 2D bands in the Raman spectra confirms the existence of graphene in the nanocomposite. The intensity ratio of D to G bands (I_D_/I_G_) is used to identify the disorder in the in-plane sp^2^ domains. I_D_/I_G_ ratio is 0.22 and this indicates a low degree of defects of graphene in the nanocomposite. Also, the intensity ratio of 2D to G bands (I_2D_/I_G_) is dependent on the number of graphene layers^51,52^. I_2D_/I_G_ ratio is 0.78 and this confirms the presence of multilayers of graphene in the nanocomposite. It is observed that there is a blue shift in the 2D band of the nanocomposite by 25 cm^−1^. This is ascribed to chemical interactions between ZnO NPs and GN^53^.

XPS is a powerful tool to analyze the chemical states and surface composition of GN/FA-ZnO nanocomposite. As shown in Fig. 5b, XPS spectra can be deconvoluted into four different peaks by applying multiple gaussian fitting. The four deconvoluted peaks exhibit carbon atoms hybridization in different functional groups, which resembles the main characteristic peaks of carbon, oxygen, and zinc. C–C bond located at 284.5 ± 0.01 eV in the functional groups corresponds to sp^2^ hybridized of carbon atoms in GN layer. Oxygen-bonded carbons demonstrated by carbon in C-O, carbonyl carbon (C=O) and carboxylate carbon (O-C=O) are located at 284.9 ± 0.07 eV, 286 ± 0.2 eV, and 289.7 ± 0.07 eV, respectively^54–56^. It is obvious that sp^2^ carbon (C–C) peak has a higher intensity, which confirms that GN layers covering ZnO. The presence of carboxylate carbon (O–C=O) bond exhibits a formation bond between oxygen of ZnO and carbon atoms located at the edges of GN layers^57^. Thus, this confirms the formation of GN/FA-ZnO nanocomposite^54^.

The crystal structure of GN/FA-ZnO nanocomposite is examined by XRD analysis. Nine well-crystalline diffraction peaks including the characteristic peak of plane (101) at 36.0° are depicted in Fig. 5c. Hexagonal (wurtzite) ZnO structure is matched with GN/FA-ZnO nanocomposite with a small shifting toward lower angles relative to the standard database (JCPDS card No. 36-1451)^58,59^. The average crystallite size (D) is estimated via Debye–Scherrer’s equation (Eq. 1)^60^:

1 $$\mathrm{D }=\frac{\mathrm{k \lambda }}{{\upbeta }_{hkl}\left(2\uptheta \right)\mathrm{cos}\left(\uptheta \right)}$$

where β_hkl_ is full width at half maximum (FWHM), k is a constant known as a shape factor of 0.89, λ is the wavelength of X-ray radiation, and θ is Bragg’s diffraction angle. The mean crystallite size of GN/FA-ZnO corresponding to the most intense peak (101) is 20.2 nm and the average crystallite size of GN/FA-ZnO is 19.0 ± 2.2 nm. Interestingly, the average crystallite size (D) can also be calculated by applying Williamson-Hall (W–H) expression (Eq. 2)^61^:

2 $${\upbeta }_{\mathrm{hkl}}\left(2\uptheta \right)\mathrm{ cos}\left(\uptheta \right)= \frac{\mathrm{k\lambda }}{\mathrm{D}}+4\mathrm{\varepsilon sin}\left(\uptheta \right)$$

where ε is the microstrain fluctuations in the crystal. As shown in Fig. 5d, D is extracted from the intercept of the W–H plot as 20.8 nm. The D calculated from the W–H plot is slightly higher than the Debye–Scherrer equation, which is attributed to existence of diverse geometries along with microstrain in d_hkl_. Additionally, ε in ZnO crystal structure are extracted from the slope of the W–H plot. Regarding the presence of GN nanosheets in GN/FA-ZnO nanocomposite, Fig. 5c exhibits the characteristic peak of GN, C (002) at 23.92° with d_hkl_ of 0.37 nm^62,63^. Furthermore, XRD pattern demonstrates that the nanocomposite also contained graphene oxide (GO) and its characteristic peak C (001) at 11.74° with d_hkl_ of 0.75 nm and another peak at 10.63° with d_hkl_ of 0.83 nm [8, 13, 14]^62,64,65^. The peak at 12.7° with d_hkl_ of 0.34 nm is attributed to reduced graphene oxide/graphene oxide nanohybrid^64^.

### Morphological properties of ZnO nanostructures

**Figure 6:**
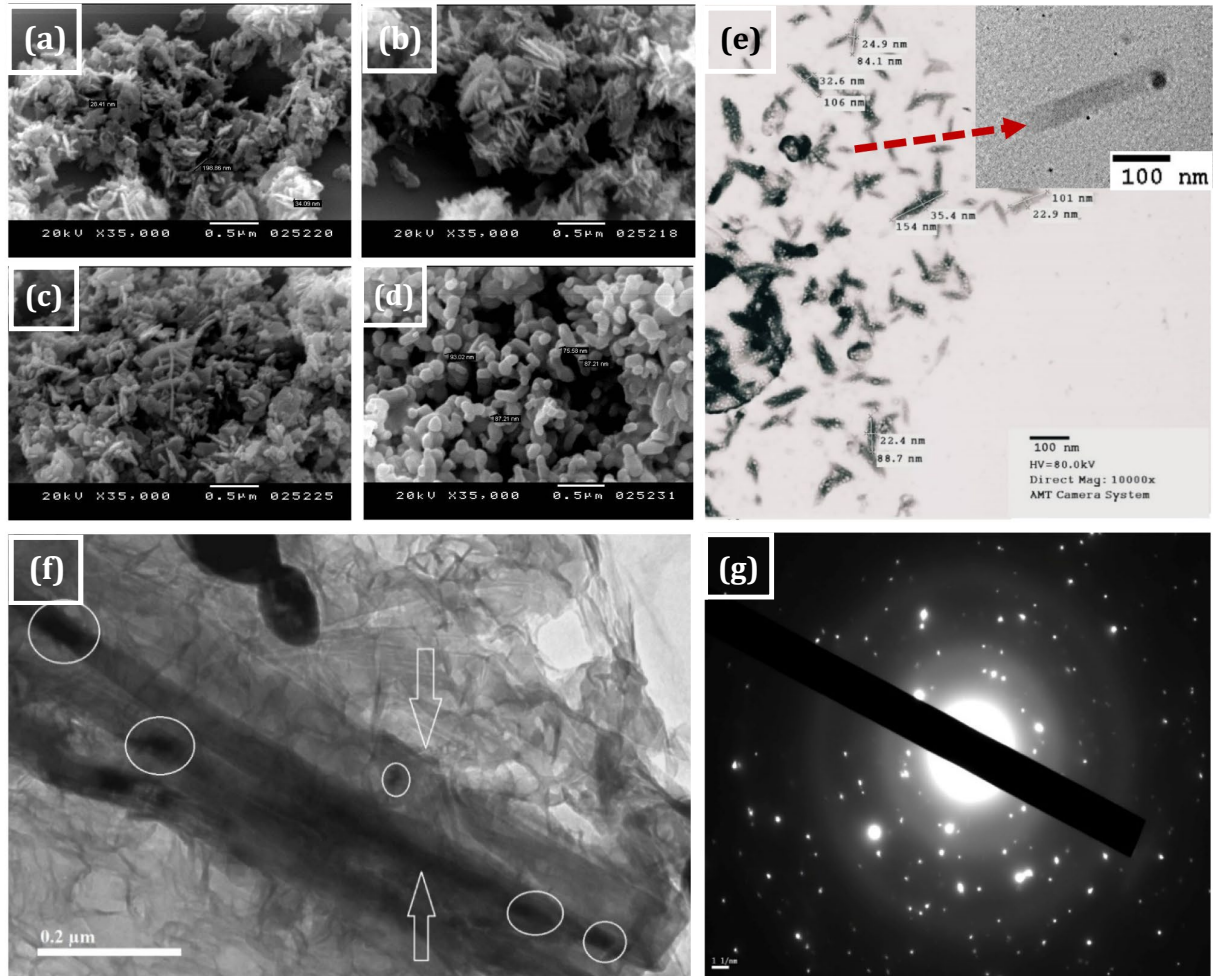
SEM images of ZnO nanostructures at different annealing temperatures (**a**) 100, (**b**) 300, (**c**) 500, (**d**) 700 °C and (**e**) SEM image of 10% GN/FA-ZnO nanocomposite, (**f**) HRTEM image of 10% GN/FA-ZnO nanocomposite; (**g**) SAED pattern of 10% GN /FA-ZnO.

SEM and HRTEM are used to illustrate the microstructures, particles size and homogeneity of FA-ZnO NPs and GN/FA-ZnO nanocomposite. SEM images of the ZnO nanostructures annealed at different temperatures are shown in Fig. 6. The morphological properties of ZnO nanostructures are changed with annealing temperatures from nanorods to nanospheres due to the variation of nucleation rate and crystal growth^66^. Annealing at 100 °C results in agglomeration of nanorods with about diameter within a range of 28.41–34.09 nm and length of 198.86 nm as shown in Fig. 6a. The transformation of this shape to homogenous nanospheres with a particle size of about 85.5 nm at annealing temperature of 700 °C is observed as illustrated in Fig. 6d.

SEM image is performed for 10% GN/FA-ZnO and displayed in Fig. 6e. The dispersed nanorods accompany with little number of spherical structures is noted for GN/FA-ZnO composite. The presence of ZnO on both sides of GN sheets reduces the Van der Waals force between the layers of GN and prevents aggregation. The nanocomposite nanorods have dimensions within a range of 22.4–35.4 nm in diameter and 84.1–154 nm in length. HRTEM image of 10% GN/FA-ZnO is illustrated in Fig. 6f which proves that the GN nanosheets and FA-ZnO NPs are successfully conjugated with each other. SAED patterns of the GN/FA-ZnO nanocomposite with well-defined diffraction spots shown in Fig. 6g indicate the polycrystalline nature of the specimen.

### Surface charges measurements

Zeta potential (ZP) is used to measure surface charges. ZP values of FA-ZnO NPs and GN/FA-ZnO nanocomposite are − 15.7 mV and − 16.3 mV, respectively. It is known that nanoparticles with ZP value between − 10 and + 10 mV are neutral, while the nanoparticles with ZP higher than + 30 mV or less than − 30 mV are strongly cationic and anionic, respectively. Consequently, FA-ZnO NPs and GN/FA-ZnO nanocomposite have intermediate dispersity and stability according to the previous reported articles^67^.

### Bioimaging experiment

**Figure 7:**
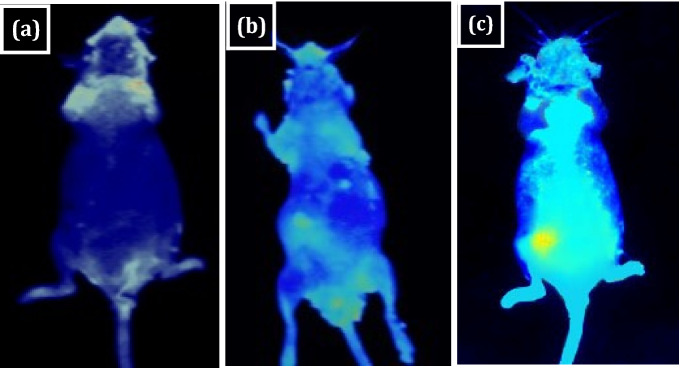
In vivo bioimaging of mice at 400 nm excitation wavelength: (**a**) bioimaging of control group; (**b**,**c**) bioimaging using GN/FA-ZnO nanocomposite.

Tumor bearing mice in vivo bioimaging experiments with and without injection of GN/FA-ZnO nanocomposite are carried out using down conversion technique. Bioimaging of tumor using Photon imager with an excitation wavelength of 400 nm is shown in Fig. 7a–c that indicates the imaging of the whole body of mice. Bright and clear signal of fluorescence are appeared after the injection in different mice during a short time that reveals the fast uptake of the nanocomposite through tumor tissue as shown in Fig. 7b,c. This indicates that GN/FA-ZnO nanocomposite fluorescence is not significantly affected by the autofluorescence of the body. Interestingly, the position of fluorescence in the tumor confirms that GN/FA-ZnO nanocomposite is successfully prepared for in vivo tumor targeting.

**Figure 8:**
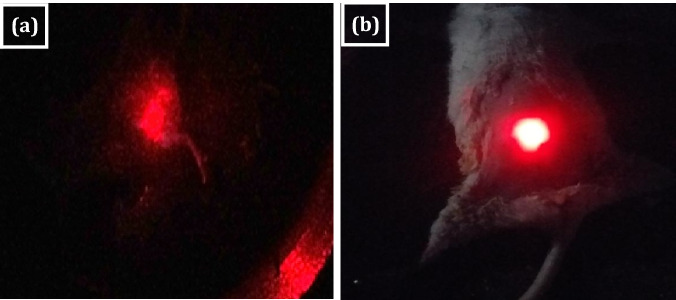
In vivo bioimaging of mice at 630 nm excitation wavelength: (**a**) bioimaging of control group; (**b**) bioimaging using 10% GN/FA-ZnO nanocomposite.

In a dark room, in vivo bioimaging of tumor with and without GN/FA-ZnO nanocomposite injection using laser with excitation wavelength 630 nm is shown in Fig. 8 using up-conversion technique. The images are recorded using a digital camera. Bright luminescent spots generating white spots in the image is observed. The white spots from GN/FA-ZnO nanocomposite are considered a physical property that is produced from by the combination of the emissions of GN/FA-ZnO nanocomposite with the tumor. The use of FA is not only for electrostatically separation but also acts as a ligand for cancer cell receptors and FA is utilized to detect tumor cells due to the presence of folate receptors in tumor cells.

## Conclusion

GN/FA-ZnO nanocomposite with a dual mode emission was successfully prepared using direct green method. It was found that GN/FA-ZnO nanocomposite has high content of GN and low contents of GO. G, D, and 2D bands in the Raman spectra confirmed the formation of GN in the nanocomposite. I_D_/I_G_ ratio was found to be 0.22 and indicated a low degree of defects of GN in the nanocomposite. I_2D_/I_G_ ratio was 0.78 and confirmed the presence of a multilayer graphene structure in the nanocomposite. ZnO nanostructues were transformed with annealing temperature from nanorods to nanospheres where ZnO annealed at 100 °C has nanorods diameter of 28.41–34.09 nm and length of 198.86 nm. The transformation of this shape to nanospheres with a particle size of 85.5 nm at annealing temperature of 700 °C was observed. From XPS, high intensity sp^2^ carbon (C–C) peak indicated GN layers covering ZnO. GN/FA-ZnO nanocomposite had wurtzite structure. Tumor bearing mice in vivo bioimaging experiments with and without injection of GN/FA-ZnO nanocomposite were carried out and the position of fluorescence in the tumor confirmed that GN/FA-ZnO nanocomposite is prepared for in vivo tumor targeting. Using up-conversion technique white spots from GN/FA-ZnO nanocomposite were recorded.

## Data Availability

The datasets used and/or analyzed during the current study available from the corresponding author on reasonable request.
